# Investigating the Features of PDO Green Hams during Salting: Insights for New Markers and Genomic Regions in Commercial Hybrid Pigs

**DOI:** 10.3390/ani11010068

**Published:** 2021-01-01

**Authors:** Martina Zappaterra, Paolo Zambonelli, Cristina Schivazappa, Nicoletta Simoncini, Roberta Virgili, Bruno Stefanon, Roberta Davoli

**Affiliations:** 1 Department of Agricultural and Food Sciences (DISTAL), University of Bologna, Viale Fanin 46, I-40127 Bologna, Italy; paolo.zambonelli@unibo.it; 2 Stazione Sperimentale per l’Industria delle Conserve Alimentari (SSICA), Viale Faustino Tanara 31/A, I-43121 Parma, Italy; cristina.schivazappa@ssica.it (C.S.); nicoletta.simoncini@ssica.it (N.S.); roberta.virgili@ssica.it (R.V.); 3 Department of Agrifood, Environmental and Animal Science, University of Udine, Via delle Scienze 208, I-33100 Udine, Italy; bruno.stefanon@uniud.it

**Keywords:** swine, genetic marker, ham processing, ham quality

## Abstract

**Simple Summary:**

In recent years, the meat industry is looking with increased interest at the implementation of non-invasive new tools for predicting the final quality of dry-cured hams and monitoring ham aging. The selection of raw meat and the control of the salting procedure to predict the quality of dry-cured hams are of primary importance for meat processors. The identification of genetic markers associated with ham traits and related to different aptitudes of the thighs towards salting phases and weight losses is a primary goal for the Italian pig production chain. This paper addresses the need for investigating the associations between genomic markers and ham traits obtained through the application of non-invasive technologies for monitoring hams before and during the salting process. To our knowledge, this is the first study investigating the markers and genes associated with ham traits obtained through the use of Ham Inspector^TM^ apparatus.

**Abstract:**

Protected Designation of Origin (PDO) dry-cured hams production is greatly dependent on raw meat quality. This study was performed to identify genetic markers associated with the quality of dry-cured ham. Carcass traits of 229 heavy pigs belonging to three commercial genetic lines were registered (weight, EUROP classification). Phenotypic traits (*Semimembranosus* muscle ultimate pH, ham weight and lean meat content, adsorbed salt) of the corresponding thighs, undergone PDO ham process in three different plants, were measured, using a fast and non-invasive technology. Green ham weight and lean meat percentage influenced the estimated salt content and the weight loss during salting, even if the processing plant greatly affected the variability of the measured ham traits. The genomic data were obtained with the GeneSeek Genomic Profiler (GGP) 70k HD Porcine Array, using the slaughter day and the sex of the animals in the statistical analyses. The phenotypic traits were associated with the genotypes through GenAbel software. The results showed that 18 SNPs located on nine porcine chromosomes were found to be associated with nine phenotypic traits, mainly related to ham weight loss during salting. New associations were found between markers in the genes *Neural Precursor Cell Expressed Developmentally Down-Regulated 9* (*NEDD9*, SSC7), *T-Cell Lymphoma Invasion and Metastasis 2* (*TIAM2*, SSC1), and the ham quality traits. After validation, these SNPs may be useful to improve the quality of thighs for the production of PDO dry-cured hams.

## 1. Introduction

The production of Protected Designation of Origin (PDO) Parma and San Daniele hams plays an economic role of primary importance in the Italian pig production chain [1] and represents a point of excellence for the Italian pork chain. The quality of the raw meat and the carcass composition greatly influence both the suitability of the thighs to obtain high-quality PDO dry-cured hams such as Parma and San Daniele [2,3]. The fresh thighs used for the production of PDO Parma hams are obtained from heavy pigs slaughtered at a live weight of at least 140 to160 kg, with an age of at least nine months, and belonging to specific selected breeds defined by Parma ham Consortium [4]. These requirements are essential for obtaining hind legs with features suitable for long curing periods; additional required parameters of the thighs are the presence of subcutaneous fat of 15 to 30 mm of thickness to minimize processing losses [2]. These features affect both the aptitude of meat to adsorb salt during the ham salting periods and the weight loss of the ham after salting during the long maturation, which may last from 12 to 24 or more months [5]. However, over time, extensive analytical surveys were carried out to determine the contribution of this food to sodium intake, and medical and public health organizations recommended to reduce sodium dietary intake to prevent hypertension and other diseases [5,6]. On the other hand, an excessive reduction in the amount of adsorbed salt can worsen the technological performances, causing an increase in proteolysis, higher softness, and the production of off-flavors due to the release of free amino acids and peptides during ripening [5,6]. The research on environmental and genetic factors involved in salt adsorption and in the sodium amount of dry-cured ham has attracted great interest based on these contrasting nutritional and technological requirements behind the issue of sodium reduction. Several factors contribute to influencing the final salt amount in dry-cured hams; among them, a crucial role is represented by the know-how of the different ham processors [5]. The ham weight and size, the inter- and intramuscular fat content, the thickness of subcutaneous fat, and the lean meat content of the hind leg represent the main factors that can influence also the aptitude of the ham to adsorb salt [7,8,9,10]. The study and identification of genes and genetic markers associated with these traits could provide new and important tools for the ham industry for improving ham yield during processing. Also the physico-chemical modifications of thigh muscles post-mortem, such as pH decline, water activity, proteolytic and lipolytic reactions, produce changes in color, taste, flavor, texture [11], and salt absorption in the hams [5]. These traits are affected, at least in part, by the animal genetic background as reported by studies indicating genetic markers associated with *Semimembranosus* muscle (SM) pH and drip loss [12,13], and with enzymes activity in San Daniele hams [14]. Most of these association studies have been performed on the purebred pig lines used in the two-way or three-way crossbreeding schemes [12,15], but few association studies exist between genetic markers and ham ripening related traits measured directly in heavy pigs used for dry-cured ham production. Moreover, the genetic improvement of quality traits of dry-cured hams is difficult because these characteristics cannot be easily measured in live animals and are cost-effective phenotypes.

In recent years, different non-invasive technologies based on X-rays or near-infrared (NIR) spectroscopy were investigated as quality control techniques in the ham industry [16]. Magnetic induction technology is among the most promising to estimate lean content in green hams [17] and the salt absorbed by processed hams [18]. These new non-invasive technologies allow for the collecting of a huge number of phenotypic data. Thus, the identification of genetic markers and new tools to assess and help to predict these traits may be of primary importance for pig production chain and ham processors [10,14,19].

This work aims to investigate the variability of green ham traits in a sample of 229 commercial hybrid heavy pigs during salting and the genomic regions associated with the measured phenotypes. These regions will be singled out through a Genome-Wide Association Study (GWAS), which will allow identifying DNA markers and candidate genes associated with phenotypic characteristics of the fresh hams measured during the first stages of the ham processing. The identified SNPs might be of interest to improve ham yield after salting and the quality of the finished product. The identification of genetic markers associated with the quality of the thighs for PDO dry-cured hams can offer new helpful tools for the highly innovative pig production chain.

## 2. Materials and Methods

### 2.1. Animal Data Availability

The samples and hams used in the present study were obtained from the slaughtering of commercial heavy pigs intended for human consumption, and thus the present research did not need approval from a research ethics committee. The heavy pig hybrids were slaughtered in commercial abattoirs in four slaughter days between March and July 2018. The animals were slaughtered in compliance with the European rules [20,21] on the protection of animals during transport and related operations and slaughtering. All slaughter procedures were monitored by the veterinary team appointed by the Italian Ministry of Health. The carcasses came from crossbred pigs reared in three different farms and were obtained from the crossbreeding of the three main breeds reared in Italy for heavy pig production, namely Large White, Landrace, and Duroc. These commercial hybrids were kept in collective pens until they reached the slaughter weight. The pigs were fed diets complying with the dietary inclusion limits established by the Parma Ham Consortium [4] and were slaughtered at an average live weight of 160 kg. Each farm sent their heavy pigs to a specific abattoir, and the thighs were then processed by a specific processing plant. Thus, the farm of farrowing, abattoir, and ham processing plants are collinear variables. Similarly, also the slaughter days match with the farms, abattoirs, and plants, with the thighs coming from the same slaughter day for the hams of plant 1, from two slaughter days for hams processed in plant 2, and from one day of slaughtering for ham processed in plant 3.

### 2.2. Carcass and Ham Traits

A total of 229 fresh hams were collected from carcasses classified as “U”, “R”, and “O” in compliance with Commission Implementing Decision, 2014/38/EU [22]. Pig carcasses were graded according to the European EUROP carcass grading system [22] which considers the lean meat content estimated for the whole carcass. In particular, carcasses classified as “U” are characterized by a lean meat content between 50% and 55%, carcasses “R” are characterized by a lean meat content between 45% and 50%, and carcasses “O” are characterized by a lean meat content between 40% and 45%. Fresh hams were elaborated at three different dry-cured ham processing plants operating in accordance with tutelary regulations of Parma ham manufacturing [23,24]. A sample of SM was collected for the genomic analyses, and frozen at −20 °C until processed for DNA extraction.

The pH of fresh hams was measured in SM with a Hamilton glass electrode probe attached to a portable pH meter (WTW pH3110, Weilheim, Germany). Homogeneous sets of hams in terms of muscle pH at 24 h post-mortem (pHu) ranged between 5.50 and 5.90 were used for each plant.

Then, all fresh hams underwent traditional salting for Italian dry-cured ham, based on a two-step addition of salt [25], following the standard procedure of each plant.

### 2.3. Non-Invasive Magnetic Induction (MI) System Analysis

The lean amount of green hams and the salt content of the salted ones were determined by the Ham Inspector^TM^ apparatus (Lenz, Barcelona, Spain). The system based on electromagnetic induction (MI) technology generates a signal with an amplitude depending on the ham lean amount and salt content, estimated using a proper calibration [17,18].

Two hundred and twenty-nine fresh hams were scanned with the MI system set in the “RAW” mode, installed at each plant. The lean amount expressed as a percentage of ham weight was estimated by using previously developed predictive models reported in Simoncini et al. [17]. This model was improved, including a greater number of dissected and analyzed hams, and estimating the new prediction accuracy (RMSE = 1.34%). After the first salting and at the end of the salting steps, the unabsorbed salt was brushed away, and the same salted hams were scanned with the MI system set in the “salted” mode. The salt content of the lean part, expressed as a percentage on a wet basis, was estimated by using a previously developed predictive model, in accordance with Schivazappa et al. [18]. Prediction accuracy of the model including a greater number of dissected and analyzed salted hams was estimated (RMSE = 0.14%).

All scanned hams were tempered at 3 ± 0.5 °C and temperature tested with the thermometer Ebro TFX 410 Pt1000 (Xylem Inc, Rye Brook, NY, USA) inserted into SM muscle (5 cm depth) to avoid possible drifts of MI signal, associated with variations in sample temperature.

The weight of the fresh hams as well as their weights during the salting process (after the first salting and at the end of the salting period) was recorded by the MI apparatus in order to calculate the corresponding weight losses, expressed as percentage loss of fresh ham weight.

### 2.4. Statistical Analyses of Ham Traits

All statistics of phenotypic traits were obtained by SPSS version 22.0 software platform (SPSS Inc., Chicago, IL, USA); normal distribution of data was investigated before statistical analyses. The boxplot procedure was applied to present the distribution of measured lean percentage of hams, differently labeled according to the EUROP grid (lean carcass grading). Data of ham traits were analyzed using Generalized Linear Model (GLM) procedure; the models included the processing plant and the sex of the pigs respectively, as fixed factors. These factors were included in the model since they are known to affect ham quality [3]. The Least Significant Difference (LSD) posthoc test was applied to compare the Estimated Marginal Means (EMMs). Finally, the Pearson’s correlation analysis was performed to investigate the relationships between all quality and technological traits of hams in each processing plant.

### 2.5. Genotyping and Association Study

The collected SM samples were used for the genomic analyses. DNA extractions were carried out using a standard protocol by an outsource laboratory (Agrotis S.r.L.-LGS, Cremona, Italy, http://www.lgscr.it/ENG/index.html) where the genotype analyses were also performed. For the genotyping, the GeneSeek^®^ Genomic Profiler-GGP-70k HD Porcine chip (Illumina, San Diego, CA, USA; https://emea.illumina.com/products/by-type/microarray-kits/ggp-porcine.html) containing 68516 SNPs was used using the procedures indicated by the company. The SNPs were mapped using *Sus scrofa* Genome Assembly Build 11.1 (NCBI: https://www.ncbi.nlm.nih.gov/assembly/GCF_000003025.6; ENSEMBL: https://www.ensembl.org/Sus_scrofa/Info/Index). The sex of the animals (females, castrated males) was obtained on the basis of the genotypes for the sex chromosomes. The genotypic data were filtered using gPLINK (version 2.050, based on PLINK version 1.07) [26] and GenAbel (version 1.8-0, run on R version 3.4.4) [27] discarding the markers or samples not passing these thresholds: all the markers with call rate < 90%, with minor allele frequency < 5%, which are not in Hardy-Weinberg equilibrium [28] (*p*-value < 0.001); all the individuals with more than 10% missing genotypes and with Identity by State (IBS) > 90%. After filtering, 54,569 SNPs and 169 individuals were retained. The large number of excluded animals (n = 60) was because their IBS was above the threshold set at 90%. The association analyses were carried out using *polygenic_hglm* and *qtscore* functions implemented on the GenAbel package according to the procedure described in Nicolazzi et al. [29]. The statistical model included the day of slaughter and the sex as predictive variables, together with the effect of the SNP and the genomic kinship matrix. The genomic kinship matrix was obtained with GenAbel and was used to estimate the relatedness between the animals. Pedigree data were not available for these animals since commercial hybrids are obtained from heterospermic artificial insemination. Also, the information concerning litters and dams was not available. These data can be available in fully controlled experimental studies, but they are difficult to obtain in commercial farm conditions, where cross-fostering is a common practice. The *p*-values of the associations between ham traits and SNPs were corrected for the “deflation” factor, as reported by GenAbel. Markers were considered significant with corrected *p*-values below the chromosome-wide significant threshold (Table S1), and the trend towards the significance threshold was set at 5.00 × 10^−5^ [30]. The markers with corrected *p*-values below these thresholds were further considered for the estimation of the genotypic effects. Their genotypes were extracted from the PED file generated through the gPLINK tool using in-house implemented scripts in the R environment [31]. Linear mixed models were performed to obtain the Estimated Least Squares Means (LSM) for the genotypes of the significant markers. The associated traits were used as dependent variables; the genotype of each marker taken individually was modeled as a predictive variable together with the fixed effects of sex and slaughter day. The linear regression model did not include the random effect of the genomic kinship matrix. For each marker found with the GWAS, the additive and dominance genetic effects were also estimated. The additive effect was estimated as half of the difference between the two homozygous groups: a = 1/2(BB − AA), with A and B that indicate the first and the second allele of the analyzed markers, respectively. The dominance effect was estimated as the difference between the heterozygous group and the average of the two homozygous groups in each locus: d = AB − 1/2(AA + BB). These analyses were performed using functions in the packages *nlme* [32], *lsmeans* [33], *lme4* [34], and *car* [35] in the R environment [31]. The genes located in the region flanking the identified markers (±500 kilobases from the associated marker) were further considered for the identification of candidate genes for the traits. The list of the flanking genes was obtained using the BioMart tool [36] and was submitted to David Bioinformatics Resources version 6.8 on-line tool (https://david.ncifcrf.gov/). Candidate genes were identified on the basis of their location (the nearest gene to the significant marker) and biological role. In order to find possible splice sites or motifs with biological relevance, the sequences flanking the intronic variants found associated with ham traits were submitted to the Tomtom tool in MEME Suite version 5.3.0 [37] (http://meme-suite.org/). The found motifs are ranked based on their Bonferroni significance (*q*-value) for the found match between the query sequence (or its complementary) and the motif in JASPAR CORE 2014 database.

## 3. Results

### 3.1. Ham Quality and Technological Traits

**Figure 1:**
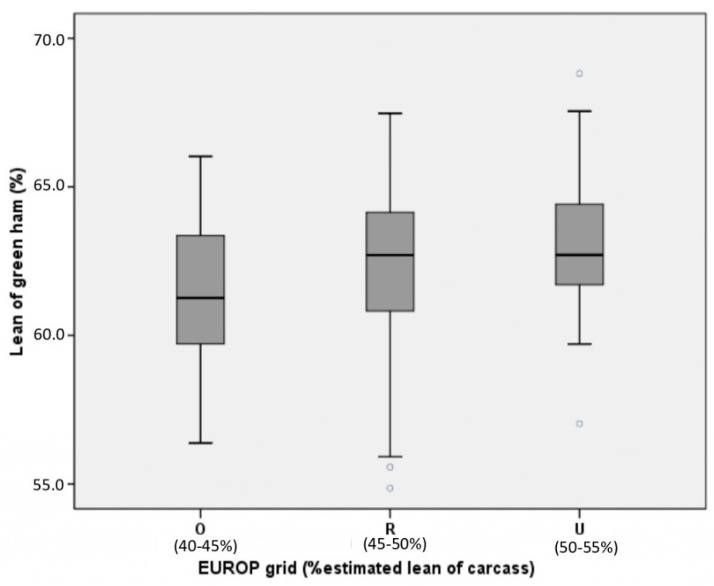
Boxplots of the lean content (%) estimated by Ham Inspector^TM^ for the green hams grouped according to the EUROP classes (U, R, and O) of the corresponding carcasses. For each EUROP class, the range of % estimated lean carcass is reported. Blank circles represent outliers.

The variability noticed for ham lean % is reported in Figure 1. The boxplot displays the distribution of the lean % of hams estimated by Ham Inspector^TM^, in relation to the lean % of the corresponding carcasses according to EUROP classification [22]. The EUROP classification of the original carcass is printed on the skin of each fresh ham supplied to dry-cured ham producers, to provide information on the lean % expected in fresh ham.

The class R, corresponding to an estimated lean % of the carcass in the range 45 to 50% is the most abundant (n = 110) compared to classes O (lean % = 40 to 45%, n = 36) and U (lean %= 50 to 55%, n = 62). In the current study, the lean % of the hams scanned by Ham Inspector^TM^ shows higher values (R = 62.3 ± 2.7, U = 63.0 ± 2.0, O = 61.6 ± 2.2) than those estimated from the EUROP classification of the corresponding whole carcass.

All fresh hams were processed in three different manufacturing plants in accordance with tutelary regulations of Parma ham [23,24].

The results of GLM analysis including “processing plant” and “sex” as fixed effects and their interaction are summarized in Table 1. For each trait, the EMMs (Estimated Marginal Means), the standard error, and the corresponding significance level are reported.

Differences in the measured ham traits between processing plants (*p* < 0.001) were detected. In particular, processing plant 1 handles the heaviest hams between the tested plants (15.1 kg), and hams leaner (62.7%) than plant 3 (60.6%). This condition affects salting weight losses, yielding higher values at the end of salting for plant 1 compared to other plants. The salt % predicted in ham lean content after both salting steps differed significantly between processing plants (*p* < 0.001), reporting greater values for plant 3 and 2, respectively (Table 1).

The sex of the pigs generated differences for the lean % of green hams (*p* < 0.001) and their weight loss after the first (*p* < 0.05) and at the end of the salting (*p* < 0.01; Table 1), and the predicted salt contents (*p* < 0.01). The higher lean % of green hams from females caused higher weight losses during salting, and higher values of salt content at the end of salting (2.60%) if compared to barrows (2.53%). As for the salt content at the end of salting, the interaction between processing plant and sex was significant (*p* < 0.05): the three plants differed regardless of the sex of the animal, but in the case of females the salt values were higher than in barrows.

The results of the Pearson’s correlation analysis performed between ham traits measured in each processing plant are reported in Tables S2–S4. The strongest and most significant correlations were found in plant 2 (Table S3): pH was negatively correlated with salt content measured at the first salting step (r = −0.241, *p* < 0.01) and the weight loss at the end of salting (r = −0.388, *p* < 0.001). The weight of green hams was negatively correlated with salt content after the first salting in all tested processing plants (Tables S2–S4), whereas, only for plant 2, the negative correlation coefficient with salt content at the end of the salting process was significant (r = −0.486, *p* < 0.001; Table S3). For all processing plants (Tables S2–S4), the lean % of green hams was positively related to ham weight losses and to the salt contents predicted after the first and at the end of the salting steps. In each processing plant, the highest positive correlation coefficients have been found between green ham weight and ham weight after the first salting (plant 1: r = 0.999, *p* < 0.001; plant 2 r = 0.999, *p* < 0.001; plant 3 r = 0.998, *p* < 0.001), green ham weight and ham weight at the end of salting (plant 1: r = 0.997, *p* < 0.001; plant 2 r = 0.999, *p* < 0.001; plant 3 r = 0.996, *p* < 0.001), and between ham weight after first and at the end of salting (plant 1: r = 0.997, *p* < 0.001; plant 2 r = 0.999, *p* < 0.001; plant 3 r = 0.999, *p* < 0.001).

### 3.2. Association Study Results

The performed GWAS allowed the identification of eight markers displaying a Bonferroni corrected *p*-value significant at the chromosome-wide level, reported in bold in Table 2. The most significant association was found for pHu, which is linked to WU_10.2_18_17949287 marker. This SNP is an intergenic variant located at 17 Mb on *Sus scrofa* chromosome 18 (SSC18). Green ham lean % showed an association with a region on SSC4 at 2 Mb, where the marker ASGA0016987 is located. This SNP is an exon variant of a non-coding transcript (ENSSSCT00000066959.1), and most of the genes comprised in the flanking region are non-coding genes. Green ham weight, ham weight after first salting, and ham weight at the end of the salting showed an association with the same two markers, namely CASI0010463 and WU_10.2_14_144250775. The first is located on SSC15 at 12 Mb, in an intron of the gene *Neurexophilin 2* (*NXPH2*). WU_10.2_14_144250775 is an intergenic variant in a region characterized by high gene density, on SSC14 at 132 Mb. Two SNPs were significant for ham weight loss after first salting: ASGA0031014, an intron variant of the gene *Neural Precursor Cell Expressed, Developmentally Down-Regulated 9* (*NEDD9*), and WU_10.2_10_74620421, a variant in a non-coding transcript on SSC10. Salt content at the end of salting showed two markers displaying a chromosome-wide significant association. The first (INRA0000796) is located on SSC1 at 13 Mb, and is an intron variant of the gene *Regulator of G Protein Signaling 17* (*RGS17*); the second (ASGA0102337) is an intron variant of the gene *GRAM Domain Containing 1B* (*GRAMD1B*), located on SSC9 at 50 Mb. Eleven markers showed less significant associations with the ham traits, with adjusted *p*-values comprised between the chromosome-wide significance level and the threshold of 5.00 × 10^−5^ (Table 2). The complete lists of the top 20 markers most associated with the measured traits are reported in File S1. Table S5 reports the complete list of all the genes located in the regions flanking the markers reported in Table 2. The results of the functional association obtained for the genes in Table S5 are reported in Table S6. No significant terms were identified, and none of them seemed to indicate a direct involvement of the candidate genes in muscle or fat development. Thus, further discussion of the obtained associations was based on the review of the scientific literature.

For each of the found markers, the LSM of the genotypes were estimated and reported in Table 3. For pHu, both markers showed genotype distributions quite unbalanced, with GG genotype poorly represented (WU_10.2_18_17949287) or not represented at all (WU_10.2_4_91195648) in our sample. Other identified markers showed a genotypic class poorly represented (*n* < 10 animals, i.e., ASGA0016987, ALGA0002237, CASI0010463, WU_10.2_7_118557013) or completely lacking (ALGA0044906, INRA0000796). The weights of fresh thighs and hams after first and at the end of salting were associated with the same markers, namely CASI0010463 and WU_10.2_14_144250775. Furthermore, the marker ASGA0026341 showed an additive genetic effect on both ham weights after first salting and at the end of salting. In fact, the AA animals for this marker showed lower ham weights than GG pigs (Table 3). Additive genetic effects were also found for H3GA0000815, WU_10.2_7_118557013, WU_10.2_14_36295226, and ASGA0031014 (Table 3). In particular, the AA animals for the H3GA0000815 SNP were associated with a lower lean % in green hams; the A allele of WU_10.2_7_118557013 was related to higher weights at the end of salting; the G allele of ASGA0031014 showed lower weight losses, and the GG animals for the WU_10.2_14_36295226 SNP presented a higher % of salt adsorbed at first salting. Concerning the ASGA0031014 marker, the three genotypic classes were well represented, following the 1:2:1 ratio.

The results obtained with MEME Suite indicated that none of the identified intronic variants were located in splice sites, but the marker ASGA0031014 fell into an intronic region of the gene *Neural Precursor Cell Expressed, Developmentally Down-Regulated 9* (*NEDD9*) harboring several binding sites recognized by *Forkhead box O* (*FOXO*) transcription factors (Table S7). The in-silico analysis indicated that the mutation ASGA0031014 changed the sequence recognized by *FOXO3* (*q*-value = 0.050; Figure S1a), *FOXO1* (*q*-value = 0.074; Figure S1b), and *FOXO4* (*q*-value = 0.074; Figure S1c). The sequences recognized by these transcription factors are the reverse complement of the region flanking ASGA0031014, since the *NEDD9* gene is located on the reverse strand (strand −1 in Table S5). For this reason, ASGA0031014 has T/C as alternate alleles in Figure S1.

## 4. Discussion

The development of new technologies enabling a fast and non-invasive measure of green ham potential to be processed into typical dry-cured ham is a goal for pig production chain and PDO ham producers. In the last decade, technologies such as those based on X-rays have been studied [16], with the aim of monitoring and gathering information in a non-invasive way on green hams intended for dry curing, focusing on their qualitative traits and the amount of salt absorbed during processing. The Ham Inspector^TM^ system permits an on-line and non-invasive inspection of the hams, a classification of thighs based on the lean content (calculated as a percentage of green ham weight), and an estimation of salt amount adsorbed in the lean part during the salting stages. The use of Ham Inspector^TM^ in three processing plants qualified for the production of PDO Parma ham provided the phenotypic data and information to perform the GWAS. At present, green hams allowed to be processed into PDO Parma hams are those labeled as U, R, and O according to the EUROP grid, with a few specific exceptions [38]. In the current study, a high variability in ham lean % within U, R, and O classes was detected. In particular, the thigh lean % estimated from Ham Inspector^TM^ was higher than the lean % of the corresponding carcasses estimated from EUROP grading. Thus, the thigh lean % given by Ham Inspector^TM^ and the carcass lean % obtained from EUROP grading provide different information. This is anyway expected, because ham is a lean cut, with a higher lean % than that of the corresponding EUROP carcass class. The measures estimated by Ham Inspector^TM^ about the lean-to-fat ratio of each thigh proved to be crucial to homogenously group hams during processing [6]. Processing plants showed differences for all the measured phenotypes, except for ham weight loss after the first salting. This result must be carefully evaluated as the on-field conditions of the present study did not permit to distinguish the specific effect of the processing plant, which included also the effects of the pig genetic type, farm management, and abattoir. Thus, the effect of the processing plant may be overestimated in the present study. The effect of the lean amount of green hams on the processing weight losses is underlined by the positive and significant correlation coefficients displayed in the three plants. This is in line with previous findings: weight losses are indeed known to be greater in hams obtained from animals with greater lean %, whereas the presence of inter, intramuscular and covering fat, containing less water than muscular tissue, is associated with a reduction of weight losses [2]. As can be expected, the lean % of green hams was strongly related to salt content after the first salting period and at the end of salting: this behavior is related to the higher salt and water diffusion coefficients of Fick’s law in lean hams than in the fat ones [39]. Currently, the variability detected in the phenotypic traits of processed ham as a consequence of the differences in raw matter and in the production plants remains in full compliance with the tutelary guidelines of Parma hams [23].

With the aim of finding genomic regions and candidate genes associated with the green ham weight, lean %, salting losses, and contents of adsorbed salt, we used sex and slaughter day (and thus also the ham processing plant) as fixed effects in the GWAS model. GWAS indicated eight markers significant at the chromosome-wide level, and eleven with a Pc1df below the threshold of 5.00 × 10^−5^. The most significant association was found between pHu and the WU_10.2_18_17949287 marker, which is located in an intergenic region of SSC18, harboring the genes *Plexin A4* (*PLXNA4*), and *Muskelin 1* (*MKLN1*). This association signal with the *PLXNA4* region could support the findings reported by Bordbar et al. [40] in Simmental beef cattle. These authors found that the bovine *PLXNA4* gene harbored 18 SNPs significantly associated with muscle development in the Simmental breed. Despite this strong association, no clear biologic evidence supporting the *PLXNA4* role in bovine muscle development was found [40]. The pHu variability was also found associated with the WU_10.2_4_91195648 marker, on SSC4. Anyway, both WU_10.2_18_17949287 and WU_10.2_4_91195648 markers showed unbalanced genotype distributions, which may have biased the association results, causing an overestimation of the marker effects.

Thigh lean % was associated with the marker H3GA0000815 located in an intronic region of the gene *T-Cell Lymphoma Invasion And Metastasis 2* (*TIAM2*). Among the identified associations, the H3GA0000815 marker showed the clearest additive effect, with the GG animals showing a higher lean % than those with the AA genotype. The lower frequency observed for the GG genotype in our samples suggests that the favorable allele G may be used in selection schemes to increase lean mass deposition in the pig breeds used for the production of PDO hams. Interestingly, the *TIAM2* gene was found in a previous study to be located in a region displaying a selective signature in a Duroc pig population [41]. This gene encodes a guanine nucleotide exchange factor with a role in intracellular signal transduction and in the regulation of cell migration and cell focal adhesions [42]. Cell migration and focal adhesions are essential steps in organogenesis, and also muscle development is known to involve a series of morphogenetic events including cell fusion, migration, and epidermal attachment [43]. Despite the fact that this result seems to agree with a previous study [41], to date, it is not possible to draw a clear hypothesis linking this gene with thigh lean % as the knowledge of the *TIAM2* gene and its roles in pig muscle development is still mostly unknown.

The variability of green ham weight, and ham weight after first salting and at the end of the salting period showed to be associated with the same markers, indicating a pleiotropic effect of these mutations. The LSM estimated for these markers were concordant among the measured traits, with the same genotypic class displaying higher estimated LSM for the weights of the fresh thigh and the ham after first salting and at the end of salting. This pattern was expected given the degree of shared variability between these three traits, as demonstrated by their high and positive phenotypic correlations, indicating that the higher the weight of the fresh thigh, the heavier are the same hams after first salting and at the end of salting period. Ham weights at the first salting and at the end of salting were significantly associated with the two markers ASGA0026341 and WU_10.2_7_118557013. An additive effect was found for both these markers, which are located in intergenic regions on SSC5 and SSC7, respectively. This is not surprising, since the literature indicates that the majority of GWAS peaks are located in non-coding or intergenic regions [44], and may therefore be a signal indicating that the found association may be due to causal mutations in closely located genes or to mutations in a still unknown gene. Among the genes located near WU_10.2_7_118557013 is *Calmodulin 1* (*CALM1*), which encodes for one of three calcium-binding calmodulin proteins. *CALM1* mediates the control of a large number of enzymes, ion channels, aquaporins, and other proteins through calcium-binding, and controls also the transport of glucose and other sugars in hepatic cells [45]. Its gene expression was also found to be downregulated in the *Longissimus* muscle of pigs with a high deposition of intramuscular fat [46], suggesting that this gene may have a role also in the metabolic pathways influencing pig meat quality.

The ham weight loss after first salting was found to be associated with ASGA0031014, on SSC7. The alleles for this marker show a significant additive effect and balanced genotype distributions in the considered sample. The most favorable genotype for this locus was GG, which displayed the lowest weight losses during processing. This mutation is an intron variant of the gene *NEDD9*, which codes for a focal adhesion protein that acts as a scaffold to regulate signaling complexes important in cell attachment, migration, and invasion [47], as well as apoptosis and the cell cycle [48]. Some studies of different authors reported that *NEDD9* is essential for the Transforming Growth Factor β (TGF-β) signaling pathway [49,50]. Moreover, members of the TGF-β superfamily can profoundly regulate mesenchymal stem cell differentiation, as well as adipogenesis and myogenesis [51,52]. Due to the involvement of *NEDD9* in TGF-β signaling, it is, therefore, possible that changes in its gene sequence may affect also TGF-β signaling and the differentiation of mesenchymal stem cells during muscle development. Furthermore, *NEDD9* is known to take part in the inhibition of primary cilia formation during embryogenesis [53]. Primary cilia are non-motile organelles that have been shown to play an important role as antennae to extracellular stimuli during embryogenesis [53], adipogenesis, and myofibrogenesis [54]. In our previous study, we found several genes related to primary cilia associated with intramuscular fat deposition [55], and *NEDD9* may take part in this complex signaling pathway related to muscle and adipose tissue development in embryogenesis. Interestingly, the marker ASGA0031014 fell into an intronic region harboring several sequences recognized by FOXO transcriptional regulators. In particular, this intronic variant is located in a site matching the motifs recognized by *FOXO1, FOXO3,* and *FOXO4* transcription regulators, suggesting that changes in this sequence may also affect the ability of those transcription regulators to recognize this binding site. This hypothesis seems to be supported in the literature, which indicated that *NEDD9* gene expression was found to be regulated by another forkhead box member (*FOXC1*) [56] and that *FOXO1* has a major role in the regulation of skeletal muscle differentiation and fiber type specification in mammals [57]. Taken together, these results suggest that ASGA0031014 may be a useful marker for the improvement of ham yield during processing. Our hypothesis needs anyway a validation with further specific studies.

In addition to these markers, other mutations were associated with the ham traits, even though they did not present significant additive effects. Among the markers associated with the ham weights at the first and at the end of the salting steps is CASI0010463, an intron variant of the *Neurexophilin 2* (*NXPH2*) gene. A Run of Homozygosity (ROH) region was found in the *NXPH2* gene in Valdostana Black Pied and Valdostana Chestnut bovine breeds [58]. This evidence seems to suggest a possible role of *NXPH2* in livestock traits related to muscle development and production efficiency. However, this gene has never been studied before in pigs and the limited knowledge of the *NXPH2* gene does not permit to reach a complete interpretation of the associations found for this gene in the present study. Another marker associated with green ham weight, ham weight after first salting, and ham weight at the end of salting is WU_10.2_14_144250775. This marker results to be unmapped in the latest release of the Ensembl database [59], while it was located at 132,664,262 bp on SSC14 in Illumina 11.1 mapping. Following the latter mapping, WU_10.2_14_144250775 is an intergenic variant. The closest genes to this marker are *Acyl-CoA Dehydrogenase Short/Branched Chain* (*ACADSB*), *H6 Family Homeobox 2* (*HMX2*), *H6 Family Homeobox 3* (*HMX3*), and *BUB3 Mitotic Checkpoint Protein* (*BUB3*). The sequence comprising *ACADSB*, *HMX2*, *HMX3*, and *BUB3* is highly conserved between mammals [60], suggesting that this region could be of primary biological importance. Anyway, no clear association exists between muscle development and deposition, and these genes. Further investigation should be dedicated to testing the role of these genes located in this SSC14 region on production traits and porcine muscle development. Also of interest is the marker ASGA0102337, an intron variant of the *GRAM Domain Containing 1B* (*GRAMD1B*) gene. This gene codes for a cholesterol transporter that mediates non-vesicular transport of cholesterol from the plasma membrane to the endoplasmic reticulum [61,62]. *GRAMD1B* gene expression in muscle was found associated with obesity in the mouse model [63], and mutations in its sequence were associated with feed efficiency in a beef cattle population with individuals from various breeds [64]. As fat deposition is important in determining salting losses and salt uptake of the green hams, mutations in the *GRAMD1B* sequence may also influence salting and maturation traits. However, the possible effect of this gene on ham quality traits needs confirmation with studies in larger samples.

## 5. Conclusions

To our knowledge, this is the first study describing significant associations between porcine candidate genes and ham traits, measured in a non-invasive way using a Magnetic Induction System. This on-line and non-invasive technology allows the estimation of green ham lean % and the salt content in the salted ones, on a great number of hams during processing. The GWAS identified several markers associated with the measured traits, and SNPs in candidate genes related to the neuromuscular junction, and muscle development during embryonic stages were found associated with lean % and ham weights. Among the markers associated with ham traits, the marker located in *NEDD9* could be of particular interest for further studies and for implementing selection schemes aimed at improving ham yields after the salting steps. Further studies including a greater number of hams and different processing conditions are needed to strengthen the investigated associations.

## Figures and Tables

**Table 1:** Effect of the processing plant (PP) and sex (S) of the animals on the traits (Estimated Marginal Means ± standard error) of hams (green and after salting steps).

Ham Traits	Processing Plant (PP)				Sex (S)			PPxS
	1	2	3	*p*-Value	Barrow	Female	*p*-Value	
	n. 60	n.120	n.50		n. 105	n. 125		*p*-Value
pH_u_	5.63^c^ ± 0.02	5.69 ^b^ ± 0.01	5.79 ^a^ ± 0.02	<0.001	5.71 ± 0.01	5.70 ± 0.01	n.s.	n.s.
Weight _GH_, kg ^1^	15.10^a^ ± 0.10	13.90 ^b^ ± 0.10	12.60 ^c^ ± 0.20	<0.001	13.90 ± 0.11	13.80 ± 0.10	n.s.	n.s.
Lean _GH_, % ^2^	62.70^a^ ± 0.30	62.90 ^a^ ± 0.20	60.60 ^b^ ± 0.30	<0.001	61.10 ± 0.20	62.90 ± 0.20	<0.001	n.s.
Weight _1S_, kg ^3^	14.90^a^ ± 0.10	13.70 ^b^ ± 0.10	12.50 ^c^ ± 0.20	<0.001	13.70 ± 0.10	13.60 ± 0.10	n.s.	n.s.
Weight Loss _1S_, % ^4^	1.31 ± 0.03	1.26 ± 0.02	1.36 ± 0.04	n.s.	1.28 ± 0.03	1.35 ± 0.02	<0.05	n.s.
Salt _1S_, % ^5^	1.06 ^c^ ± 0.01	1.21 ^b^ ± 0.01	1.46 ^a^ ± 0.02	<0.001	1.22 ± 0.01	1.27 ± 0.01	<0.001	n.s.
Weight _ES,_ kg ^6^	14.60 ^a^ ± 0.10	13.50 ^b^ ± 0.10	12.20 ^c^ ± 0.20	<0.001	13.70 ± 0.10	13.40 ± 0.10	n.s.	n.s.
Weight Loss _ES_, % ^7^	3.13 ^a^ ± 0.06	2.64 ^b^ ± 0.04	2.72 ^b^ ± 0.07	<0.001	2.73 ± 0.05	2.93 ± 0.04	<0.01	n.s.
Salt _ES_, % ^8^	2.37 ^c^ ± 0.02	2.80 ^a^ ± 0.02	2.52 ^b^ ± 0.03	<0.001	2.53 ± 0.02	2.60 ± 0.02	<0.01	<0.05
Salt _1S_ /Salt _ES,_ % ^9^	44.70 ^b^ ± 0.30	43.00 ^c^ ± 0.20	57.50 ^a^ ± 0.40	<0.001	48.50 ± 0.30	48.30 ± 0.20	n.s.	n.s.

Estimated Marginal Means with different letters along rows are significantly different (LSD posthoc test); n.s: not significant. ^1^ Weight of green hams measured with Ham Inspector^TM^ and expressed in kg. ^2^ Lean content of green hams estimated by Ham Inspector^TM^ and expressed as a percentage of green ham weight (%). ^3^ Weight of hams at 1st salting measured with Ham Inspector^TM^ and expressed in kg. ^4^ Weight loss measured after 1st salting and expressed as percentage loss of green ham weight (%). ^5^ Salt (as NaCl) content of the lean part of salted hams at 1st salting, estimated by Ham Inspector^TM^ and expressed as a percentage of wet weight (%). ^6^ Weight of hams measured at the end of salting with Ham Inspector^TM^ and expressed in kg. ^7^ Weight loss measured at the end of salting and expressed as percentage loss of green ham weight (%). ^8^ Salt (as NaCl) content of the lean part of salted hams at the end of salting, estimated by Ham Inspector^TM^ and expressed as a percentage of wet weight (%). ^9^ Ratio between salt at 1st salting step and salt content at the end of salting, expressed as percentage (%).

**Table 2:** The list of the significant markers, with the associated traits, the marker position, the type of variant, and the list of the candidate genes in the regions flanking the significant markers. Chromosome-wide significant markers are in bold.

Trait	Marker	Marker Rs Code	Position ^1^	MAF ^2^	Pc1df ^3^	Type of Variant	Candidate Genes in the Region ^4^
pH_u_	WU_10.2_18_17949287	rs321317414	18:17,103,785	0.22	**2.54 × 10^−6^**	intergenic variant	*PLXNA4*, *MKLN1*
	WU_10.2_4_91195648	rs342976952	4:83,519,869	0.13	3.94 × 10^−5^	intron variant of the gene *CD247*	*DCAF6, MPC2, ADCY10, MPZL1, RCSD1, CREG1, CD247, POU2F1, DUSP27, GPA33, MAEL*
Lean _GH_, % ^5^	ASGA0016987	rs80994554	4:2,020,990	0.25	**1.42 × 10^−5^**	exon variant of a non-coding transcript	*ADGRB1*, *MROH5*, *PTP4A3*, *GPR20*, *SLC45A4*
	ALGA0002237	rs80883186	1:30,193,313	0.16	3.07 × 10^−5^	intergenic variant	*SGK1, SLC2A12, TBPL1, TCF21, EYA4*
	H3GA0000815	rs80848905	1:11,662,820	0.35	4.72 × 10^−5^	intron variant of the gene *TIAM2*	*NOX3, TFB1M, TIAM2, SCAF8*
	ASGA0001055	rs80923830	1:11,684,450	0.33	4.72 × 10^−5^	intron variant of the gene *TIAM2*	*NOX3, TFB1M, TIAM2, SCAF8*
Weight _GH_, kg ^6^	CASI0010463	rs335635913	15:12,997,362	0.13	**5.48 × 10^−6^**	intron variant of the gene *NXPH2*	*NXPH2*, *SPOPL*
	WU_10.2_14_144250775	rs343048625	14:132,664,262	0.40	**1.33 × 10^−5^**	intergenic variant	*AWN*, *PSP-II*, *SPMI*, *PSTK*, *IKZF5*, *ACADSB*, *HMX2*, *HMX3*, *BUB3*
Weight _1S_, kg ^7^	CASI0010463	rs335635913	15:12,997,362	0.13	**4.13 × 10^−6^**	intron variant of the gene *NXPH2*	*NXPH2*, *SPOPL*
	WU_10.2_14_144250775	rs343048625	14:132,664,262	0.40	**1.26 × 10^−5^**	intergenic variant	*AWN*, *PSP-II*, *SPMI*, *PSTK*, *IKZF5*, *ACADSB*, *HMX2*, *HMX3*, *BUB3*
	ASGA0026341	rs80859829	5: 74,704,457	0.33	4.57 × 10^−5^	intergenic variant	*ADAMTS20, PUS7L, IRAK4, TWF1, U6, TMEM117*
Weight _ES,_ kg ^8^	CASI0010463	rs335635913	15:12,997,362	0.13	**2.15 × 10^−6^**	intron variant of the gene *NXPH2*	*NXPH2*, *SPOPL*
	WU_10.2_14_144250775	rs343048625	14:132,664,262	0.40	**4.27 × 10^−6^**	intergenic variant	*AWN*, *PSP-II*, *SPMI*, *PSTK*, *IKZF5*, *ACADSB*, *HMX2*, *HMX3*, *BUB3*
	ASGA0026341	rs80859829	5: 74,704,457	0.33	2.23 × 10^−5^	intergenic variant	*ADAMTS20, PUS7L, IRAK4, TWF1, U6, TMEM117*
	WU_10.2_7_118557013	rs325887861	7:111,991,773	0.12	4.25 × 10^−5^	intergenic variant	*EFCAB11, TDP1, KCNK13, PSMC1, NRDE2, CALM1*
	ALGA0044906	rs80886909	7:112,364,405	0.08	4.66 × 10^−5^	intron variant of the gene *TTC7B*	*PSMC1, NRDE2, CALM1, TTC7B, RPS6K, A5TTC7B*
Weight Loss _1S_, % ^9^	ASGA0031014	rs80963318	7:8,081,734	0.47	**1.00 × 10^−5^**	intron variant of the gene *NEDD9*	*GCM2*, *ELOVL2*, *SMIM13*, *NEDD9*, *TMEM170B*, *ADTRP*, *HIVEP1*
	WU_10.2_10_74620421	-	10:67,919,711	0.25	**1.57 × 10^−5^**	sequence variant of a non-coding gene	*ADARB2*
Weight Loss _ES_, % ^10^	ALGA0022599	rs80917191	4:6,665,856	0.27	2.90 × 10^−5^	intergenic variant	*KHDRBS3*
Salt _1S_, % ^11^	WU_10.2_14_36295226	rs323879154	14:34,218,184	0.44	1.71 × 10^−5^	intron variant of the genes *SUDS3* and *SRRM4*	*SUDS3, TAOK3, VSIG10, WSB2, RFC5, KSR2SUDS3, SRRM4*
	ASGA0000817	rs80921216	1:8,287,008	0.42	2.22 × 10^−5^	intergenic variant	*FNDC1, TAGAP, RSPH3, EZR, SYTL3, DYNLT1, TMEM181*
Salt _ES_, % ^12^	INRA0000796	rs332490862	1:13,370,639	0.08	**4.36 × 10^−6^**	intron variant of the gene *RGS17*	*RGS17,* *MTRFL1, FBXO5, VIP, MYCT1*
	ASGA0102337	rs81323631	9:50,457,899	0.35	**1.46 × 10^−5^**	intron variant of the gene *GRAMD1B*	*HSPA8, CLMP, GRAMD1B, SCN3B*, ZNF202, *OR6X1, OR6M1, OR4D5, OR6T1,*

^1^ The marker position in *Sus scrofa* Genome Assembly Build 11.1 is reported as *Sus scrofa* chromosome: nucleotide position in base pairs. ^2^ Minor Allele Frequency. ^3^ Significance estimates obtained with GenABEL with the correction for inflation factor (stratification effects). Bold values indicate markers significant at the chromosome-wide level. ^4^ The flanking region is the genomic region located between the marker position ± 500,000 nucleotides. ^5^ Lean content of green hams estimated by Ham Inspector^TM^ and expressed as a percentage of green ham weight (%). ^6^ Weight of green hams measured with Ham Inspector^TM^ and expressed in kg. ^7^ Weight of hams at 1st salting measured with Ham Inspector^TM^ and expressed in kg. ^8^ Weight of hams at the end of salting measured with Ham Inspector^TM^ and expressed in kg. ^9^ Weight loss measured after 1st salting and expressed as percentage loss of green ham weight (%). ^10^ Weight loss measured at the end of salting and expressed as percentage loss of green ham weight (%). ^11^ Salt (as NaCl) content of the lean part of salted hams at 1st salting, estimated by Ham Inspector^TM^ and expressed as percentage of wet weight (%). ^12^ Salt (as NaCl) content of the lean part of salted hams at the end of salting, estimated by Ham Inspector^TM^ and expressed as a percentage of wet weight (%).

**Table 3:** Estimated Least Squares Means (LSM) ± standard errors (S.E.), additive and dominance genetic effects of the markers associated with ham traits. Between brackets are reported the numbers of the observations used for each genotype.

Trait	Marker	Allele		LSM ± S.E (N)			Additive Effect	Dominance Effect
		1	2	11	12	22		
pH_u_	WU_10.2_18_17949287	G	A	5.71 a ± 0.04	5.73 a ± 0.02	5.70 a ± 0.01	n.s.	n.s.
				(10)	(55)	(104)		
	WU_10.2_4_91195648	G	A	-	5.69 a ± 0.02	5.72 a ± 0.01	-	-
				(0)	(43)	(126)		
Lean _GH_, % ^1^	ASGA0016987	G	A	63.91 a ± 0.81	61.91 b ± 0.27	62.61 ab ± 0.23	n.s.	0.042
				(8)	(64)	(93)		
	ALGA0002237	C	A	62.15 a ± 0.21	63.04 a ± 0.33	62.47 a ± 1.10	n.s.	n.s.
				(115)	(46)	(4)		
	H3GA0000815	G	A	63.57 a ± 0.48	62.71 a ± 0.25	61.75 b ± 0.26	0.0005	n.s.
				(20)	(75)	(70)		
	ASGA0001055	G	A	61.72 a ± 0.51	62.61 a ± 0.26	62.40 a ± 0.25	n.s.	n.s.
				(19)	(70)	(76)		
Weight _GH_, kg ^2^	CASI0010463	C	A	13.58 ab ± 0.68	14.05 a ± 0.16	13.66 b ± 0.09	n.s.	n.s.
				(2)	(38)	(125)		
	WU_10.2_14_144250775	G	A	13.93 ab ± 0.20	13.88 a ± 0.10	13.48 b ± 0.13	n.s.	n.s.
				(24)	(85)	(56)		
Weight _1S_, kg ^3^	CASI0010463	C	A	13.42 ab ± 0.69	13.85 a ± 0.16	13.48 b ± 0.09	n.s.	n.s.
				(2)	(37)	(123)		
	WU_10.2_14_144250775	G	A	13.74 ab ± 0.20	13.69 a ± 0.11	13.29 b ± 0.13	n.s.	n.s.
				(24)	(83)	(55)		
	ASGA0026341	G	A	14.01 a ± 0.24	13.71 a ± 0.11	13.29 b ± 0.12	0.009	n.s.
				(16)	(78)	(68)		
Weight _ES_, kg ^4^	CASI0010463	C	A	13.12 ab ± 0.69	13.63 a ± 0.17	13.24 b ± 0.09	n.s.	n.s.
				(2)	(37)	(116)		
	WU_10.2_14_144250775	G	A	13.48 ab ± 0.21	13.48 a ± 0.11	13.04 b ± 0.13	n.s.	n.s.
				(23)	(79)	(52)		
	ASGA0026341	G	A	13.70 a ± 0.25	13.50 a ± 0.11	13.05 b ± 0.12	0.022	n.s.
				(16)	(78)	(68)		
	WU_10.2_7_118557013	G	A	13.18 c ± 0.09	13.74 b ± 0.17	15.19 a ± 0.67	0.003	n.s.
				(120)	(33)	(2)		
	ALGA0044906	G	A	13.24 b ± 0.08	13.81 a ± 0.20	-	-	-
				(131)	(24)	(0)		
Weight Loss _1S_, % ^5^	ASGA0031014	G	A				1.23 b ± 0.04 (42)	n.s.
	WU_10.2_10_74620421	G	A	1.24 b ± 0.02	1.37 a ± 0.03	1.35 ab ± 0.08	n.s.	n.s.
				(91)	(61)	(10)		
Weight Loss _ES_, % ^6^	ALGA0022599	G	A	2.82 a ± 0.04	2.81 a ± 0.05	2.90 a ± 0.11	n.s.	n.s.
				(86)	(57)	(12)		
Salt _1S_, % ^7^	WU_10.2_14_36295226	G	A	1.26 a ± 0.02	1.25 ab ± 0.01	1.21 b ± 0.01	0.036	n.s.
				(28)	(85)	(49)		
	ASGA0000817	G	A	1.25 a ± 0.01	1.23 a ± 0.01	1.24 a ± 0.02	n.s.	n.s.
				(51)	(87)	(24)		
Salt _ES_, % ^8^	INRA0000796	C	A	-	2.62 a ± 0.03	2.63 a ± 0.01	-	-
				(0)	(21)	(134)		
	ASGA0102337	G	A	2.62 a ± 0.04	2.62 a ± 0.02	2.64 a ± 0.02	n.s.	n.s.
				(17)	(73)	(65)		

LSM with different letters along rows are significantly different for *p* < 0.10 adjusted with the Tukey test. “-” means that additive and dominance effect cannot be estimated for that marker as two genotypes were found in the samples. n.s. stands for not significant. ^1^ Lean content of green hams estimated by Ham Inspector^TM^ and expressed as a percentage of green ham weight (%). ^2^ Weight of green hams measured with Ham Inspector^TM^ and expressed in kg. ^3^ Weight of hams at 1st salting measured with Ham Inspector^TM^ and expressed in kg. ^4^ Weight of hams at the end of salting measured with Ham Inspector^TM^ and expressed in kg. ^5^ Weight loss measured after 1st salting and expressed as percentage loss of green ham weight (%). ^6^ Weight loss measured at the end of salting and expressed as percentage loss of green ham weight (%). ^7^ Salt (as NaCl) content of the lean part of salted hams at 1st salting, estimated by Ham Inspector^TM^ and expressed as a percentage of wet weight (%). ^8^ Salt (as NaCl) content of the lean part of salted hams at the end of salting, estimated by Ham Inspector^TM^ and expressed as a percentage of wet weight (%).

## Data Availability

The data presented in this study are available on request from the corresponding authors. The data are not publicly available due to confidentiality agreements. Supporting data can be made available to bona fide researchers subject to a non-disclosure agreement.

## Supplementary Materials

The following are available online at https://www.mdpi.com/2076-2615/11/1/68/s1, Table S1: The Chromosome-wide thresholds for significance calculated for each chromosome; Table S2: Pearson’s correlation coefficients (above the diagonal) and corresponding *p*-values (below the diagonal) between processing and analytical traits of hams (green and after 1st and at the end of salting steps) in Plant 1; Table S3: Pearson’s correlation coefficients (above the diagonal) and corresponding *p*-values (below the diagonal) between processing and analytical traits of hams (green and after 1st and at the end of salting steps) in Plant 2; Table S4: Pearson’s correlation coefficients (above the diagonal) and corresponding *p*-values (below the diagonal) between processing and analytical traits of hams (green and after 1st and at the end of salting steps) in Plant 3; Table S5: List of the genes in the regions flanking the markers associated with ham traits; Table S6: Results of the functional enrichment analysis performed on the list of the genes located in the regions flanking the markers associated with ham traits; Table S7: Results of the Tomtom tool with the binding sites recognized by different transcription regulators on the sequence flanking the marker ASGA0031014; Figure S1: The sequence flanking the marker ASGA0031014 (position 17) is recognized by the transcription factors (**a**) *Forkhead box O 3* (*FOXO3*; upper sequence), with the alternate allele C (or G if we consider the complementary DNA sequence) possibly changing the binding site recognized by *FOXO3* (*q*-value = 0.050); (**b**) *Forkhead box O 1* (*FOXO1*; upper sequence),with the alternate allele C (or G if we consider the complementary DNA sequence) possibly changing the binding site recognized by *FOXO1* (*q*-value = 0.074); (**c**) *Forkhead box O 4* (*FOXO4;* upper sequence), with the alternate allele C (or G if we consider the complementary DNA sequence) possibly changing the binding site recognized by *FOXO4* (*q*-value = 0.074); File S1: Tables with the top 20 markers associated with each ham trait and their complete output information obtained with GenAbel package.
