# Supplementary material for: Investigating the Features of PDO Green Hams during Salting: Insights for New Markers and Genomic Regions in Commercial Hybrid Pigs
Source: Animals (Basel). 2021 Jan 1;11(1):68. doi: 10.3390/ani11010068 (PMC7823679; doi:10.3390/ani11010068)
Supplement: Supplementary file 1 [file animals-11-00068-s001.zip › Figure S1.docx]

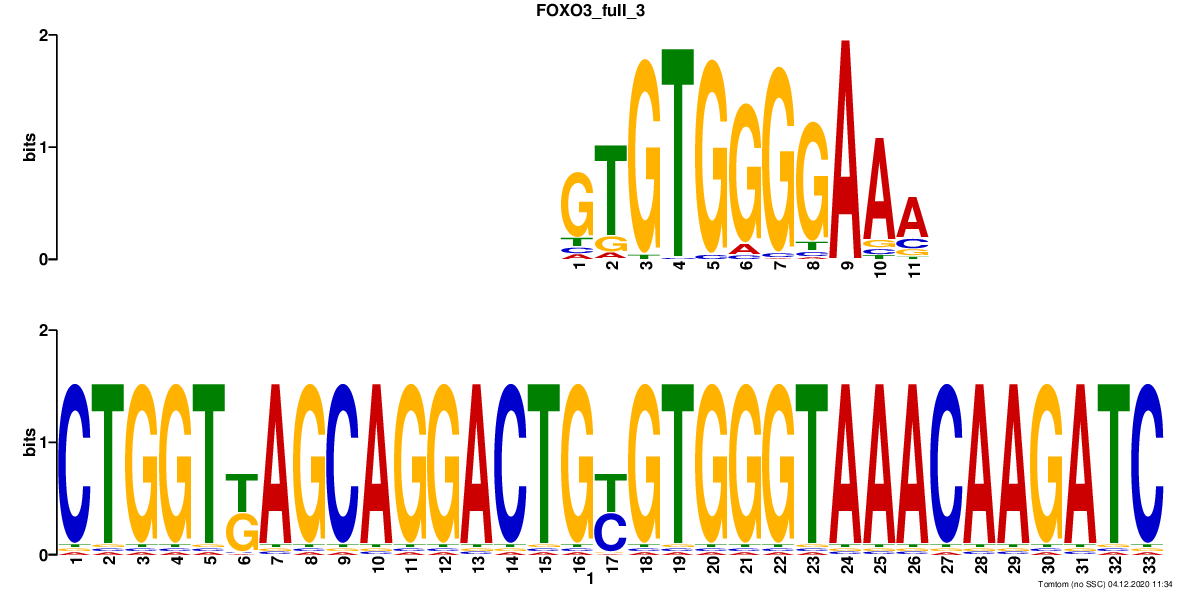


**(a)**


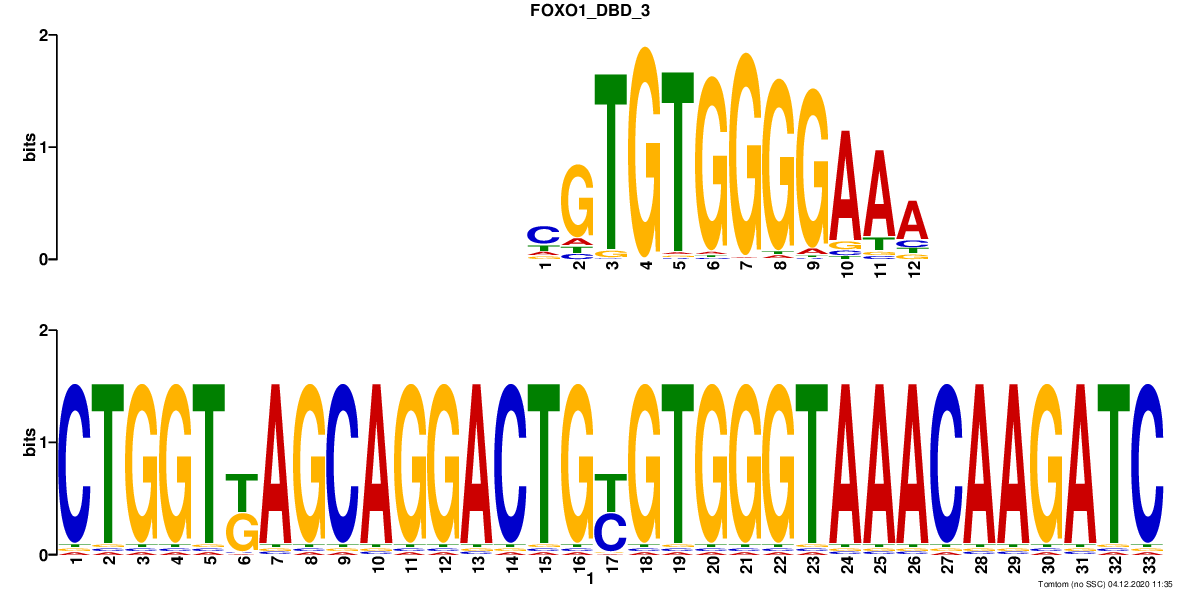


**(b)**


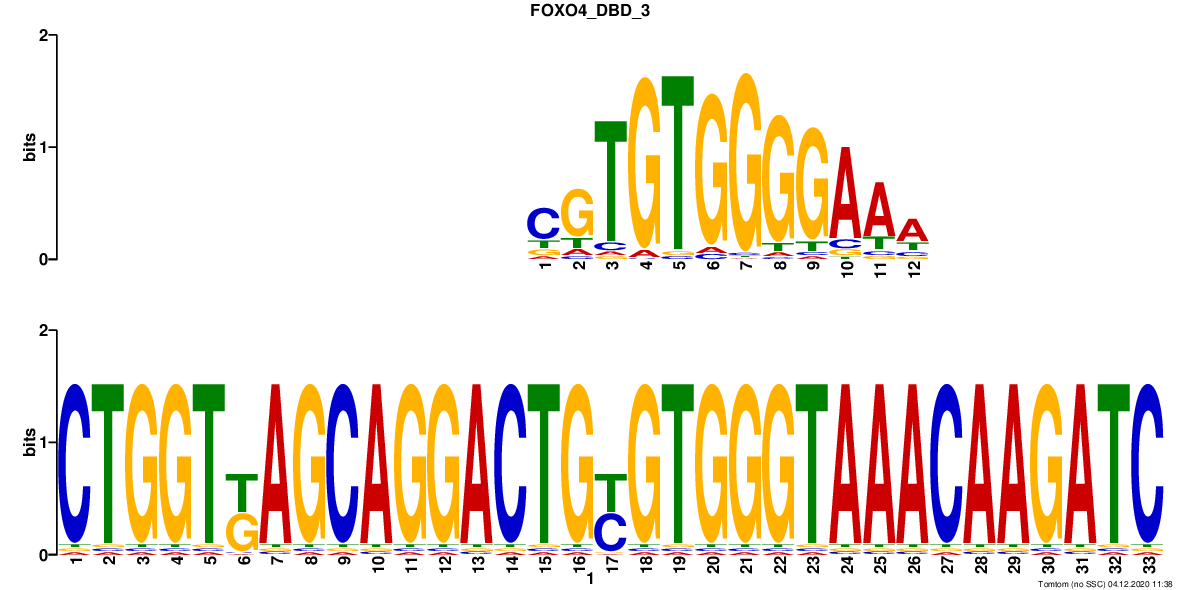


**(c)**

**Figure S1.** The sequence flanking the marker ASGA0031014 (position 17) is recognized by the transcription factors **(a)** *Forkhead box O* *3* (*FOXO3;* upper sequence), with the alternate allele C (or G if we consider the complementary DNA sequence) possibly changing the binding site recognized by *FOXO3* (q-value = 0.050); **(b)** *Forkhead box O* *1* (*FOXO1*; upper sequence),with the alternate allele C (or G if we consider the complementary DNA sequence) possibly changing the binding site recognized by *FOXO1* (q-value = 0.074); **(c)** *Forkhead box O* *4* (*FOXO4;* upper sequence), with the alternate allele C (or G if we consider the complementary DNA sequence) possibly changing the binding site recognized by *FOXO4* (q-value = 0.074).
