# Supplementary material for: Investigating the Features of PDO Green Hams during Salting: Insights for New Markers and Genomic Regions in Commercial Hybrid Pigs
Source: Animals (Basel). 2021 Jan 1;11(1):68. doi: 10.3390/ani11010068 (PMC7823679; doi:10.3390/ani11010068)
Supplement: Supplementary file 1 [file animals-11-00068-s001.zip › Table S2.docx]

**Table S2.** Pearson’s correlation coefficients (above the diagonal) and corresponding *P*-values (below the diagonal) between processing and analytical traits of hams (green and after 1st and at the end of salting steps) in Plant 1. Significant correlation coefficients are in bold.

| **Ham traits** | **pH_u_** | **Weight _GH_, kg^1^** | **Lean _GH_, %^2^** | **Weight _1S_, kg^3^** | **Weight Loss _1S_, %^4^** | **Salt _1S_, % ^5^** | **Weight _ES,_ kg ^6^** | **Weight Loss _ES_, % ^7^** | **Salt _ES_, % ^8^** |
| --- | --- | --- | --- | --- | --- | --- | --- | --- | --- |
| pH_u_ | - | -0.087 | -0.106 | 0.013 | 0.051 | -0.100 | -0.021 | -0.021 | -0.091 |
| Weight _GH_, kg^1^ | n.s. | - | **0.397** | **0.999** | 0.019 | **-0.301** | **0.997** | -0.127 | -0.068 |
| Lean _GH_, %^2^ | n.s. | <0.01 | - | **0.383** | **0.359** | **0.506** | **0.405** | **0.319** | **0.596** |
| Weight _1S_, kg^3^ | n.s. | <0.001 | <0.01 | - | -0.018 | **-0.315** | **0.997** | -0.143 | -0.081 |
| Weight Loss _1S_, %^4^ | n.s. | n.s. | <0.01 | n.s. | - | **0.374** | 0.017 | **0.427** | **0.347** |
| Salt _1S_, % ^5^ | n.s. | <0.05 | <0.001 | <0.05 | <0.01 | - | **-0.316** | **0.378** | **0.906** |
| Weight _ES,_ kg ^6^ | n.s. | <0.001 | <0.01 | <0.001 | n.s. | <0.05 | - | -0.205 | -0.085 |
| Weight Loss _ES_, %^7^ | n.s. | n.s. | <0.05 | n.s. | <0.001 | <0.01 | n.s. | - | 0.236 |
| Salt _ES_, % ^8^ | n.s. | n.s. | <0.001 | n.s. | <0.01 | <0.001 | n.s. | n.s. | - |

^1^ Weight of green hams measured with Ham Inspector^TM^ and expressed in kg.

^2^ Lean content of green hams estimated by Ham Inspector^TM^ and expressed as percentage on green ham weight (%).

^3^ Weight of hams at 1^st^ salting measured with Ham Inspector^TM^ and expressed in kg.

^4^ Weight loss measured after 1^st^ salting and expressed as percentage loss of green ham weight (%).

^5^ Salt (as NaCl) content of the lean part of salted hams at 1^st^ salting, estimated by Ham Inspector^TM^_._ and expressed as percentage on a wet basis (%).

^6^ Weight of hams at the end of salting measured with Ham Inspector^TM^ and expressed in kg.

^7^ Weight loss measured at the end of salting and expressed as percentage loss of green ham weight (%).

^8^ Salt (as NaCl) content of the lean part of salted hams at the end of salting, estimated by Ham Inspector^TM^_._ and expressed as percentage on a wet basis (%).
